# The impact of splenectomy on outcomes after distal and total pancreatectomy

**DOI:** 10.1186/1477-7819-5-61

**Published:** 2007-06-02

**Authors:** Ilias Koukoutsis, Appou Tamijmarane, Riccardo Bellagamba, Simon Bramhall, John Buckels, Darius Mirza

**Affiliations:** 1 Hepatobiliary and Liver Transplant Unit, Queen Elizabeth Hospital, Birmingham, UK

## Abstract

**Background:**

Several authors advocate spleen preserving distal pancreatectomy, because of the increased complication rate after splenectomy.

**Methods:**

Postoperative complications and survival after distal and total pancreatectomy, were recorded and retrospectively analyzed according to spleen preservation. Patients, who underwent distal and total pancreatectomy without histologically proven adenocarcinoma, or extrapancreatic disease, were included in the cohort which was divided into splenectomy and no splenectomy groups. Statistical analysis was performed using Fisher's test.

**Results:**

The study group consisted of 62 patients who underwent distal and total pancreatectomy between 26/11/1987 to 6/1/2006. Splenectomy was performed in 35 out of 62 patients (56.5%), distal pancreatectomy was performed in 49 out of 62 patients (79%). Morbidity rate was 28.6% in splenectomy group and 14.8% in the no splenectomy group (p = 0.235), while 30 days mortality rate was 2.9%; one patient died in the splenectomy group (p = 1).

**Conclusion:**

Spleen-preservation did not influence the outcomes after distal and total pancreatectomy in our series.

## Background

Pancreatectomy may be accompanied with splenectomy in distal and total pancreatic resections. Elective peripheral pancreatectomy is safer than pancreaticoduodenectomy, but carries a high morbidity rate [1-4]; intraabdominal abscess, intraabdominal hemorrhage and pancreatic fistula are the main causes [5-9]. In the past decade splenectomy was associated with increased septic complications rate [10,11]. Furthermore, several authors [12-15], suggested spleen preserving distal pancreatectomy in order to reduce postoperative septic complications[16]. The technique of spleen preserving distal pancreatectomy and its absolute and relative contraindications have been described elsewhere [3,13,17,18]. Few retrospective studies have analyzed the influence of splenectomy in the postoperative course after distal pancreatectomy, while one study has analyzed this relationship after total pancreatectomy [3,19,20]. These studies included patients with benign diseases [21]; mainly with chronic pancreatitis [3]; only with chronic pancreatitis [22]; with malignant and benign diseases [9,23] ; mainly with pancreatic trauma [19] and only with adenocarcinoma [20].

In our study, postoperative complications after distal and total pancreatectomy, were recorded and analyzed according to spleen preservation, in patients with pancreatitis (chronic and acute), benign neoplasms and other benign diseases.

## Patients and methods

Prospective collected data were retrospectively analyzed for patients who underwent distal or total pancreatectomy with or without splenectomy between 28^th ^of November 1987 and 6^th ^of January 2006. Patients with histologically proven adenocarcinoma, patients with cystadenocarcinoma, patients who underwent completion pancreatectomy after postoperative complication of pancreaticoduodenectomy, patients who underwent pancreatectomy because of abdominal trauma, patients who had hepatic metastases in laparotomy, patients who had cancer in the pancreatic head or lower common bile duct and patients who had additional procedures such as gastrectomy and colectomy were excluded from the study. The patients were divided into splenectomy and no splenectomy group. The following parameters were recorded and analyzed for each of the above mentioned groups: sepsis (SIRS and MODS), acute renal failure, pulmonary complications (atelectasia, pneumonia, pleural effusion), ARDS (acute onset, bilateral infiltrates on chest radiography, pulmonary-artery wedge pressure ≤ 18 mm Hg or the absence of clinical evidence of left atrial hypertension, acute lung injury considered to be present if PaO_2 _:FiO_2 _is ≤ 300 Acute respiratory distress syndrome considered to be present if PaO_2 _:FiO_2 _is ≤ 200), cardiac complications (atrial fibrillation, dysarrythmia), central nervous system complications (confusion, stroke), intra abdominal abscess (defined as an infected fluid collection identified by CT or ultrasound scan-guided needle aspiration and microbiologic culture), postoperative primary intra abdominal hemorrhage (1ry IA, diagnosed by the presence of fresh blood through the drains or by hypovolemic shock and abdominal distension in patients without drains), postoperative primary gastrointestinal hemorrhage (1ry GI), delayed gastric emptying, wound infection, wound dehiscence, first 30 postoperative days mortality.

Statistical analysis was performed using Fisher's two-tailed test, in the "Statistical Package for the Social Sciences" version 12 for Windows (SPSS^®^, Chicago, IL, USA). A p value less than 0.05 was considered significant.

## Results

Hospital data included 160 patients who underwent distal and total pancreatectomy between 28^th ^of November 1987 and 6^th ^of January 2006. Histologically proven adenocarcinoma had 31 patients, 20 patients had additional procedures, 13 patients had liver or peritoneal metastases at laparotomy, 11 patients had cystadenocarcinoma, 5 patients underwent laparotomy for abdominal trauma and 17 underwent surgery for other non benign diseases (data available but not shown). After fulfilling the exclusion criteria, our study group consisted of the rest 62 patients who underwent total and distal pancreatectomy with or without splenectomy. The demography, types of operations and final diagnoses are shown in Tables 1 and 2. Splenectomy was performed in 35 out of 62 patients (56.5%), distal pancreatectomy was performed in 49 out of 62 patients (79%). Morbidity rate was 28.6% (10 patients) in splenectomy group and 14.8% (4 patients) in the no splenectomy group (p = 0.235). According the type of surgery, the morbidity rate was 24.1% in distal pancreatectomy with splenectomy and 15% in distal pancreatectomy without splenectomy (p = 0.496), while in total pancreatectomy with or without splenectomy was 50% and 14.3% respectively (p = 0.266).

**Table 1:** Study group characteristics and types of operations.

	**N (%)**
**Sex**	
Male	30 (48,4%)
Female	32 (51,6%)
**Age (years)**	
Median	52
Range	22–87
**Type of pancreatectomies**	
Distal+splenectomy	29
Total+splenectomy	6
Distal (spleen preservation)	20
Total (spleen preservation)	7

**Table 2:** Final diagnoses after pancreatic resection in the total of 62 patients.

**Final diagnoses**	**Frequency**
Pancreatitis	
Chronic	19
Acute	6
Benign Neoplasms	
Adenoma	1
Papilloma	1
Insulinoma	5
Gastrinoma	1
Pseudopapillary tumor	4
Other benign diseases	
Benign neuroendocrine tumor	4
Cystadenoma	14
Pseudocyst	7

### Splenectomy vs no splenectomy group

Using the Fisher's test no studied factor was correlated with splenectomy vs no splenectomy groups (Table 3). Interestingly all the patients with postoperative sepsis were in the splenectomy group, but the difference was not statistically significant (p = 0.250).

**Table 3:** Analysis of complications in splenectomy and no splenectomy groups.

**Complications**	**Splenectomy group (n)**	**No splenectomy group (n)**	**p**	**Complications**	**Splenectomy group (n)**	**No splenectomy group (n)**	**p**
**Sepsis**	3	0	**0.250**	**Renal failure**	1	0	**1**
**Pulmonary**	1	0	**1**	DGE*	2	0	**0.5**
**Intrabdominal abscess**	1	1	**1**	1ry IA *	1	0	**1**
**Wound infection**	1	2	**0.575**	**Cardiac**	0	0	**-**
**Wound dehiscense**	1	1	**1**	CNS*	1	0	**1**
**ARDS**	1	0	**1**	1ry GI*	0	1	**0.435**

*(1ry IA = primary intra abdominal haemhorrage, 1ry GI = primary gatrointestinal haemhorrage, CNS = Central Nervous System, DGE = Delayed gastric emptying).

The two cases of intra abdominal access were treated by the radiologist with a CT guided drainage. The only case of primary postoperative hemorrhage needed a reintervention after a failed embolization.

### 30 days mortality analysis

The 30 days mortality rate in the study group of patients was 2.9%. One patient died (stroke) in the splenectomy group (p = 1). There was not recorded any postoperative death due to postsplenectomy sepsis.

## Discussion

Spleen preserving distal pancreatectomy has been advocated by many authors, because of splenectomy associated immunologic alterations and septic postoperative complications [13,16,24,25]. Holdsworth *et al*., [15] and Benoist *et al*., [21] studied patients with benign diseases and Sledzianowski *et al*., [26] studied patients with malignant and benign diseases, failed to prove the importance of spleen preservation in distal pancreatectomy, supporting the fact that the risk of overwhelming postsplenectomy sepsis in adult population with benign disease is very low (0.28%–1.9% with a 2.2% mortality rate) [15,27]. Aldridge *et al*., [3] in a group of patients with chronic pancreatitis concluded that postoperative course was similar after distal pancreatectomy regardless of splenectomy. Richardson and Scott- Conner [19] demonstrated that spleen preservation did not increase the complications rate after distal pancreatectomy. However, the study group was small (21 patients), and mainly consisted of trauma patients who underwent major additional procedures in most of the cases. Schwarz et al., [20] studied the outcomes in a group of patients (326 patients, 37 underwent splenectomy) with adenocarcinoma after distal and total pancreatectomy with or without splenectomy, concluded that splenectomy was a statistically significant unfavorable prognostic factor in survival, but not in postoperative morbidity. Shoup *et al*., [23], in a cohort with benign and low-grade malignant diseases (125 patients), reported that spleen preserving distal pancreatectomy is associated with lower infectious complications rate and reduced hospital stay, than the distal pancreatectomy with splenectomy (p = 0.01 and p < 0.01 respectively). To our knowledge, there is no other series, studying the relation of spleen preservation with all the postoperative parameters we recorded together, after distal and total pancreatectomy. Infectious complications including wound and pulmonary complications, intra abdominal abscess formation and sepsis were not statistically significant associated with splenectomy. There was no significant obvious predilection in the selection of distal or total pancreatectomy and splenectomy or spleen preservation. The mortality rate recorded in our cohort is similar to the reported in some studies [1,2] and lower than the published in other articles [3,4,28] after distal pancreatectomy. In our series there was not statistically significant difference recorded in morbidity, in the first 30 postoperative days mortality (p = 0.592 respectively). In order to fully assess the influence of splenectomy on survival after distal and total pancreatectomy, future studies including larger series of patients are required.

## Conclusion

The authors conclude that spleen preservation does not influence the outcome after distal or total pancreatectomy, in benign diseases and selected benign neoplasms.

## Competing interests

The author(s) declare that they have no competing interests.

## Authors' contributions

**IK**: Designed the study, performed bibliographic research, drafted and revised the manuscript.

**AT**: Carried out the data and participated in the writing process.

**RB**: Participated in the design of the study, helped to draft the manuscriptand and performed the statistical analysis.

**SB**, **JB**, **DM**: Participated in manuscript revision process.

All authors read and approved the final manuscript.
